# Association of oral anticoagulation persistence and health-related quality of life in stroke survivors with atrial fibrillation: long-term results of the Berlin atrial fibrillation registry

**DOI:** 10.1186/s42466-026-00534-z

**Published:** 2026-09-16

**Authors:** Lena Steindorf-Sabath, Manuel C. Olma, Serdar Tütüncü, Claudia Kunze, Ulrike Grittner, Jil Heege, Joanna Dietzel, Johannes Schurig, Peter U. Heuschmann, Matthias Endres, Karl Georg Haeusler

**Affiliations:** 1 Department of Neurology with Experimental Neurology, Charité - Universitätsmedizin Berlin, Berlin, Germany; 2 Center for Stroke Research Berlin, Charité - Universitätsmedizin Berlin, Berlin, Germany; 3 Department of Neurology, Alexianer St. Josefs-Krankenhaus Potsdam, Potsdam, Germany; 4 Institute for Biometry and Clinical Epidemiology, Charité - Universitätsmedizin Berlin, Berlin, Germany; 5 Berlin Institute of Health, Charité - Universitätsmedizin Berlin, Berlin, Germany; 6 Institute of Social Medicine, Epidemiology and Health Economics, Charité - Universitätsmedizin Berlin, Berlin, Germany; 7 Clinical Trial Center Würzburg, University Hospital Würzburg, Würzburg, Germany; 8 Institute of Clinical Epidemiology and Biometry, University of Würzburg, Würzburg, Germany; 9 Institute for Medical Data Science, University Hospital Würzburg, Würzburg, Germany; 10 German Center for Neurodegenerative Diseases Partner Site Berlin, Berlin, Germany; 11 German Centre for Cardiovascular Diseases Partner Site Berlin, Berlin, Germany; 12 German Center for Mental Health (DZPG) Partner Site Berlin, Berlin, Germany; 13 Department of Neurology, University of Ulm, Ulm, Germany; 14 Medizinische Hochschule Brandenburg Theodor Fontane, Neuruppin, Germany

**Keywords:** Stroke, Atrial fibrillation, Health-related quality of life, Oral anticoagulation

## Abstract

**Background:**

Persistence to oral anticoagulation (OAC) is crucial to prevent recurrent stroke in patients with atrial fibrillation (AF). Little is known about the association of OAC intake, persistence to OAC, and type of OAC with health-related quality of life (HRQoL) in stroke survivors with AF.

**Methods:**

This retrospective analysis of the multicenter Berlin Atrial Fibrillation Registry included 1,042 patients with acute ischemic stroke or transient ischemic attack (TIA) and AF. HRQoL data were measured at 3,12,24 and 36 months after stroke/TIA using the EQ-5D-3 L questionnaire (including EQ-Index and EQ-VAS). Associations between OAC status, persistence to and type of OAC (direct oral anticoagulants (DOACs) against vitamin K antagonists (VKAs)) at 12 and up to 36 months with HRQoL were examined using multivariable linear models.

**Results:**

Overall, 842 patients completed the 12-months follow-up and 437 patients the 36-months follow-up. After 12 months, OAC intake was associated with higher EQ-VAS scores (β = 6.70, *p* = 0.017), although this effect was not observed up to 36 months (ß= -3.67, *p* = 0.075). Non-persistence to OAC was associated with lower EQ-VAS scores at 12 (ß= -6.25, *p* = 0.004) and 36 months (ß= -5.39, *p* < 0.001). No evidenve of a statistically significant difference in HRQoL was found between patients persistent to DOACs and those persistent to VKAs. EQ-Index values were not statistically significantly affected.

**Conclusion:**

Non-persistence to OAC was associated with reduced EQ-VAS up to 36 months after stroke. Notably, no evidence of a statistically significant difference regarding HRQOL was found between patients persistent on DOAC or VKA. Ensuring long-term persistence to OAC may support HRQoL in stroke survivors with AF.

**Supplementary Information:**

The online version contains supplementary material available at https://doi.org/10.1186/s42466-026-00534-z.

## Introduction

Oral anticoagulation (OAC) is standard for secondary prevention in patients with atrial fibrillation (AF) after ischemic stroke or transient ischemic attack (TIA) [1]. Non-persistence to OAC is associated with an increased risk of stroke [2] and cognitive decline [3]. There are only a few absolute contraindications for OAC, such as active bleeding, severe thrombocytopenia, and recent intracranial hemorrhage [1]. Both, vitamin K antagonists (VKAs) and direct oral anticoagulants (DOACs) are effective in reducing the AF-related incidence of ischemic stroke [4]. In comparison to VKAs, DOACs offer a broader therapeutic window, lower inter- and intraindividual variability regarding the dose-effect relationship, and lower drug interactions [5]. As a result, guidelines recommend preferred use of DOACs over VKAs [1]. In clinical trials, the main focus has traditionally been on the efficacy and safety of OAC, with Health-related Quality of Life (HRQoL) receiving limited attention [6]. However, HRQoL is increasingly acknowledged as a meaningful outcome that reflects the patient’s perspective on treatment burden, daily functioning, and well-being - particularly in chronic conditions such as AF, where long-term OAC therapy can impact everyday life and overall HRQoL [6]. HRQoL in patients with AF is associated with increased risk for hospitalization, cardiovascular events and all-cause mortality, making it essential to identify factors associated with lower HRQoL [7–9]. The impact of type of OAC on patient-reported outcomes remained controversial, particularly regarding the potential superiority of DOACs over VKAs in terms of HRQoL [10]. Based on current evidence [11, 12], a potential HRQoL benefit of DOACs over VKAs (both Warfarin and Phenprocoumon) was hypothesized. Notably, only one study has examined the impact of OAC in stroke and TIA patients with first detected AF, reporting higher HRQoL at 12 months post-stroke/TIA in those receiving OAC compared to those without OAC [13]. To our knowledge, no studies are published examining the relationship between OAC persistence and HRQoL in patients with known or newly-diagnosed AF after stroke or TIA.

Therefore, this retrospective analysis of the multicenter, prospective *Berlin Atrial Fibrillation Registry* aimed to evaluate the association between HRQoL and OAC intake, OAC persistence and type of OAC (DOACs vs. Phenprocoumon) in patients with ischemic stroke or TIA over a 36-months follow-up period.

## Methods

### Study design and patient population

The *Berlin Atrial Fibrillation Registry* was an investigator-initiated, prospective, multicenter study. The study was sponsored by the Charité-Universitätsmedizin Berlin. The Berlin Atrial Fibrillation Registry received approval by the ethics committees of all participating sites, with initial approval by the Charité Ethics Committee, Berlin, Germany (EA2_033_14). The registry was registered with ClinicalTrials.gov on December 3, 2014 (ClinicalTrials.gov ID: NCT02306824). The study design was published previously [14]. All study patients provided written informed consent, and when the study follow-up was extended to 36 months, all participants were required to give renewed consent to continue to the study. Patients with acute ischemic stroke or TIA were eligible for inclusion, if they had a known AF or a first-detected AF during hospitalization after the index stroke/TIA. The primary outcome was patient-reported persistence to OAC (DOAC or VKA) in stroke patients with AF at 12 months post-stroke/TIA [14]. Within the VKA group, all analyses were based on phenprocoumon. Patients were classified as persistent, if the OAC prescription was documented at two consecutive time points (including discharge medication) and no interruption of ≥ 7 days was reported by the patient. An interruption of ≥ 7 days at any time during the entire observation period was sufficient to classify a patient as non-persistent, if no intake of different OACs was reported. In our analysis, switching between VKA and DOAC was considered to be persistent to OAC therapy. In treatment-specific analyses, switching between different DOACs was considered to be persistent in DOAC analysis, whereas switching from VKA to DOAC was classified as non-persistence in the VKA analysis but as persistence for DOAC analysis, if no interruption of ≥ 7 days had occurred and the DOAC was continued during upcoming follow-ups, if applicable. The seven-day threshold was used as a pragmatic study-specific definition. Only patients who had ever received the respective treatment (OAC/DOAC/VKA) were included in the OAC analysis, therefore the total number of patients may differ between the treatment-specific analyses and the so-called overall OAC analyses.

This secondary analysis aimed to analyze (i) whether the OAC status, (ii) patient-reported persistence to OAC and, (iii) the type of OAC (DOAC vs. VKA), altered HRQoL over the follow-up period.

### Outcome measures: health-related quality of life

HRQoL was assessed using the official German version (v.1.1, 1995) of the generic EQ-5D-3 L questionnaire, with scoring based on a valuation set derived from a representative German population [15]. The EQ-5D-3 L includes the EQ-5D descriptive system and the EQ-5D visual analogue scale (EQ-VAS) [16]. The EQ-5D-3 L descriptive system addresses five aspects of health: mobility, self-care, usual activities, pain/discomfort, and anxiety/depression. Each dimension has three levels (“no problems,” “some problems,” “extreme problems”). The health states can be summarized into a single value (EQ-index) from − 1 to 1, with 1 represents perfect health, and negative values indicate states considered worse than death. The EQ-VAS reflects the patient’s self-assessment of their health status with scores range from 100 (excellent health) to 0 (poorest health) [16]. In this study, HRQOL was measured at 3-, 12-, 24- and 36-month follow-up. The final question of the EQ-5D questionnaire asked whether the patient could answer the questions unreservedly; if responses were provided by proxies, the EQ-5D data were excluded. Because the residuals of the EQ-index were not normally distributed, the EQ-index was first rescaled to a 0–1 range and then transformed using the formular: (EQ-index × 100)^4^/1,000,000. Following the transformation, the EQ-index and EQ-VAS were both scaled from 0 to 100 following the approach we applied in an earlier study [13].

### Statistical analysis

A complete-case analysis was performed, including patients with no missing data for the variables included in the analysis. *p* Values < 0.05 were considered statistically significant. The three main research questions as well as a direct comparison between DOAC and VKA were analyzed using multivariable, linear models. The independent variables used for model adjustment are listed in the supplementary material, including frequency of serious adverse events (Table S1). For the 12-months follow-up, linear regression models without random effects were applied. A time series analysis for the 36-month follow-up was conducted using a linear mixed-effects model with random effects (random intercept and random slope for time at the patient level). Only variables with a minimum occurrence of 10 cases (e.g., serious adverse events) were included in the analysis to ensure statistical robustness. We further applied a joint model to account for deaths occurring between the 12- and 36-month follow-up. This approach allowed us to jointly model the longitudinal trajectory of HRQoL and the time-to-event data, thereby addressing potential bias due to mortality. Due to the exploratory nature of this project, no corrections were applied for multiple comparisons. All statistical analysis were done using R (version 4.4.1).

## Results

### Patient characteristics

**Fig. 1:**
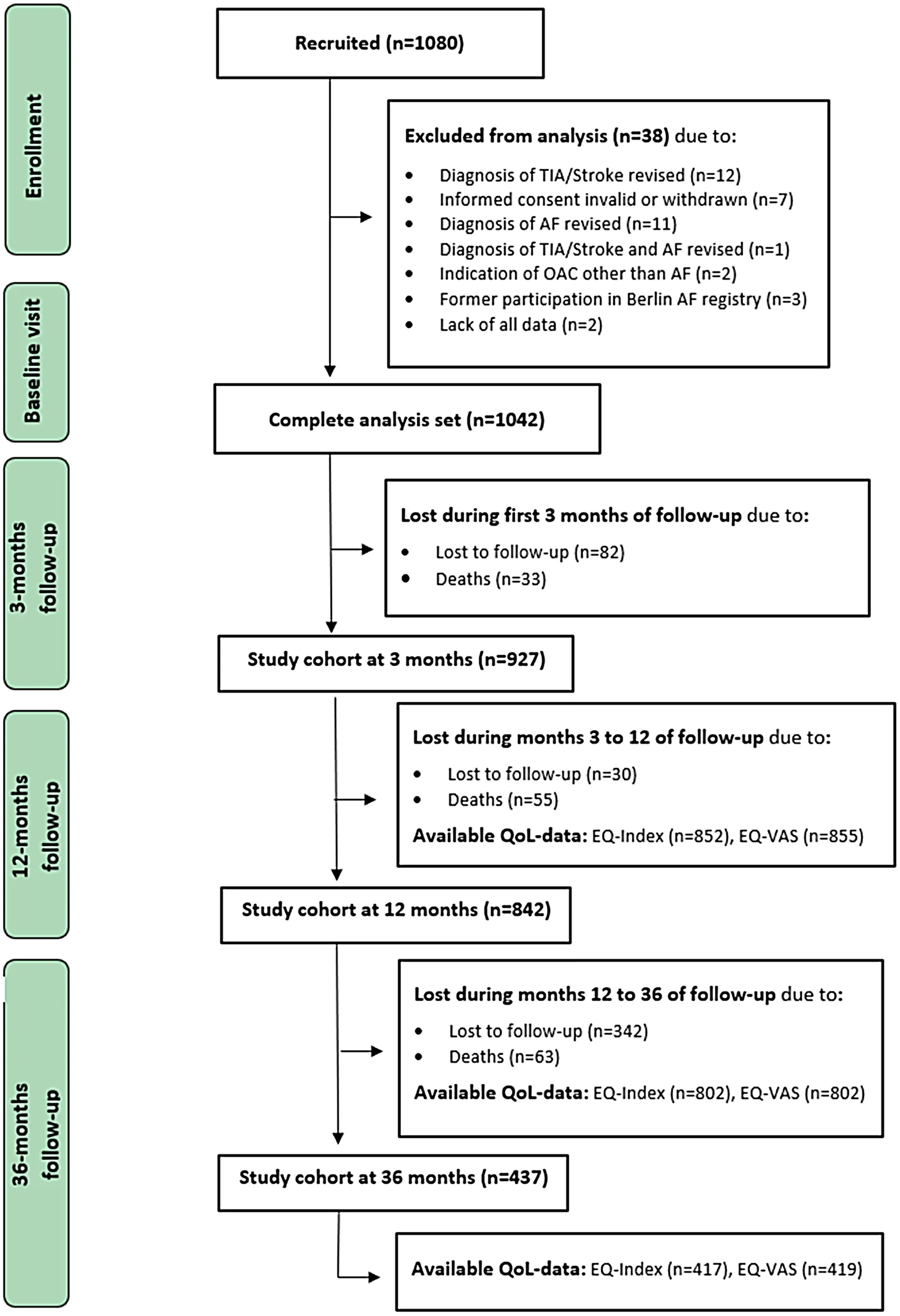
Derivation of study population

Overall, 1042 patients were included. Mean age at study inclusion was 76 years, 48.0% were female and 24.1% had a TIA as the index event (Table S2a). The median NIHSS score on admission was 2 points and the median mRS score at discharge was 2. AF was previously known in 72.7% and first diagnosed during the hospital stay in 27.3% of the patients [14]. A total of 927 registry patients were included in this retrospective analysis, as patients with lost-to-follow-up and death within three months after baseline visit were excluded (Fig. 1). Models were adjusted for stroke versus TIA as the index event leading to study inclusion; no significant differences were observed (Table S4-S12). At the 12-month follow-up, 798/842 (94.9%) patients were on OAC. Among the patients eligible for OAC persistence analysis (i.e., with OAC use in a previous follow-up visit), 744/817 (91.1%) were persistent. At the 36-months follow-up, 416/437 (95.2%) patients were on OAC. Persistence remained high at the 36-month follow-up: 375/429 (87.4%) patients were persistent with OAC. In total, 320/388 patients (82.5%) were persistent with DOACs, while 50/100 patients (50.0%) were persistent with VKAs at the 36-months follow-up.

### OAC status and health-related quality of life

**Fig. 2:**
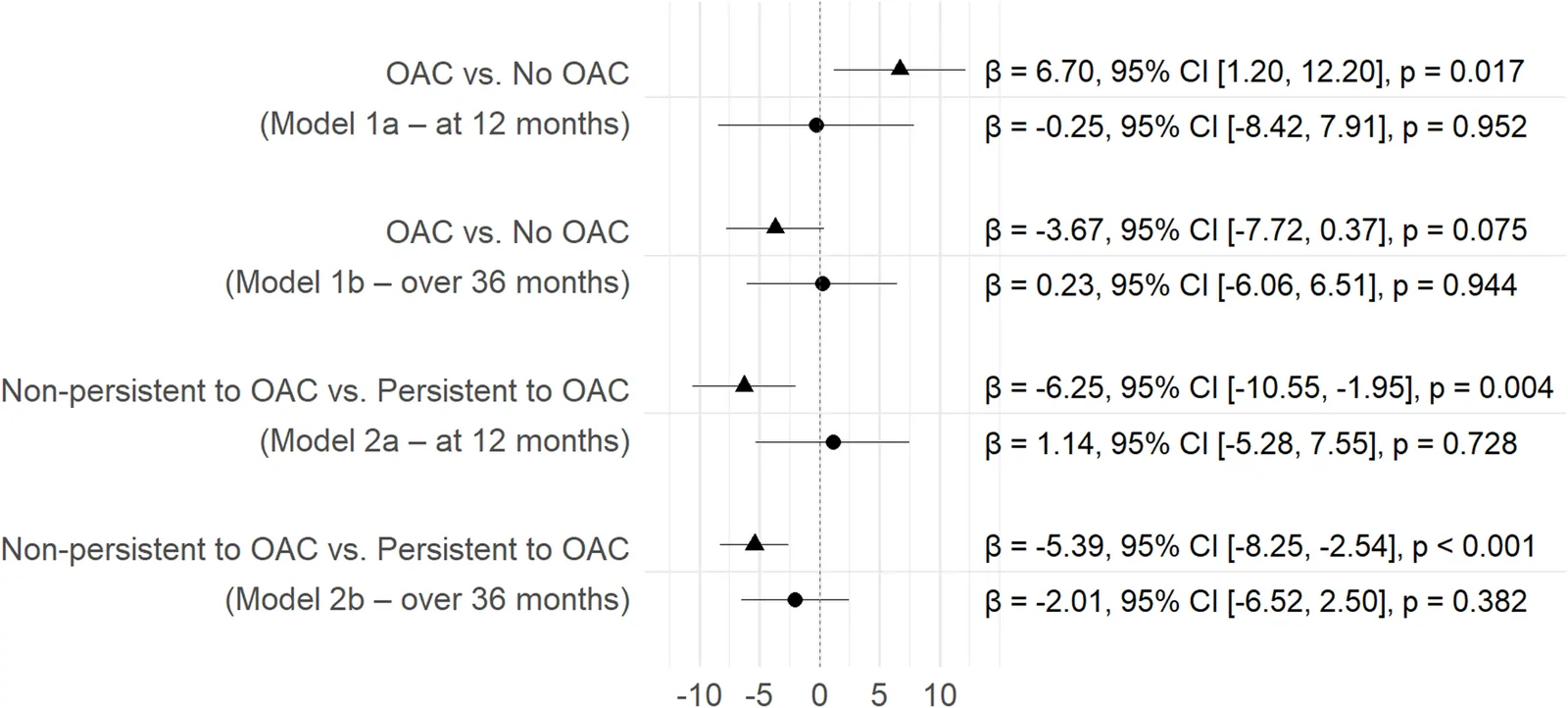
Forest plot with β estimates adjusted for covariates as specified in the respective multivariable models (see Table S4-S7) and corresponding 95% confidence intervals on EQ-Index (●) and EQ-VAS (▲). *OAC = oral anticoagulation; CI = confidence interval*

Data availability on EQ-5D ranged across the follow up time points from 90.4-95.4% (Table S3a). Unadjusted values of EQ-Index and EQ-VAS across all follow-up timepoints, stratified by OAC intake, are presented in Table S3a. Table S3b displays the transformed EQ-Index values used in multivariable analyses. Multivariable analysis revealed that OAC intake was associated with statistically significantly higher EQ-VAS scores at 12 months after the index event (β = 6.70, 95% CI [1.20, 12.20], p = 0.017; Figure 2, Table S4 – Model 1a). However, this association was not observed at 36 months (β = –3.67, 95% CI [–7.72, 0.37], p = 0.075; Figure 2, Table S6 – Model 1b).

### OAC persistence and health-related quality of life

Compared to non-persistent patients at 12-months follow-up, patients persistent to OAC were more often female, had lower mRS scores at discharge, received more often intravenous thrombolysis and endovascular treatment, and had less cardiovascular comorbidities, except of renal impairment (Table S2b). EQ-VAS scores also differed statistically significantly between patients with persistent and non-persistent OAC use. At 12 months, non-persistence was associated with lower EQ-VAS values (β = − 6.25, 95% CI [–10.55, − 1.95], *p* = 0.004; Fig. 2, Table S5 – Model 2a), and this effect remained statistically significant up to 36 months (β = − 5.39, 95% CI [–8.25, − 2.54], *p* < 0.001; Fig. 2, Table S7 – Model 2b). Notably, the negative association of non-persistence remained unchanged after accounting for deaths between the 12- and 36-month follow-up (Table S8). These effects were observed for EQ-VAS only and not for EQ-Index (Fig. 2, Tables S4–S7).

### Association of type of OAC with EQ-VAS

**Fig. 3:**
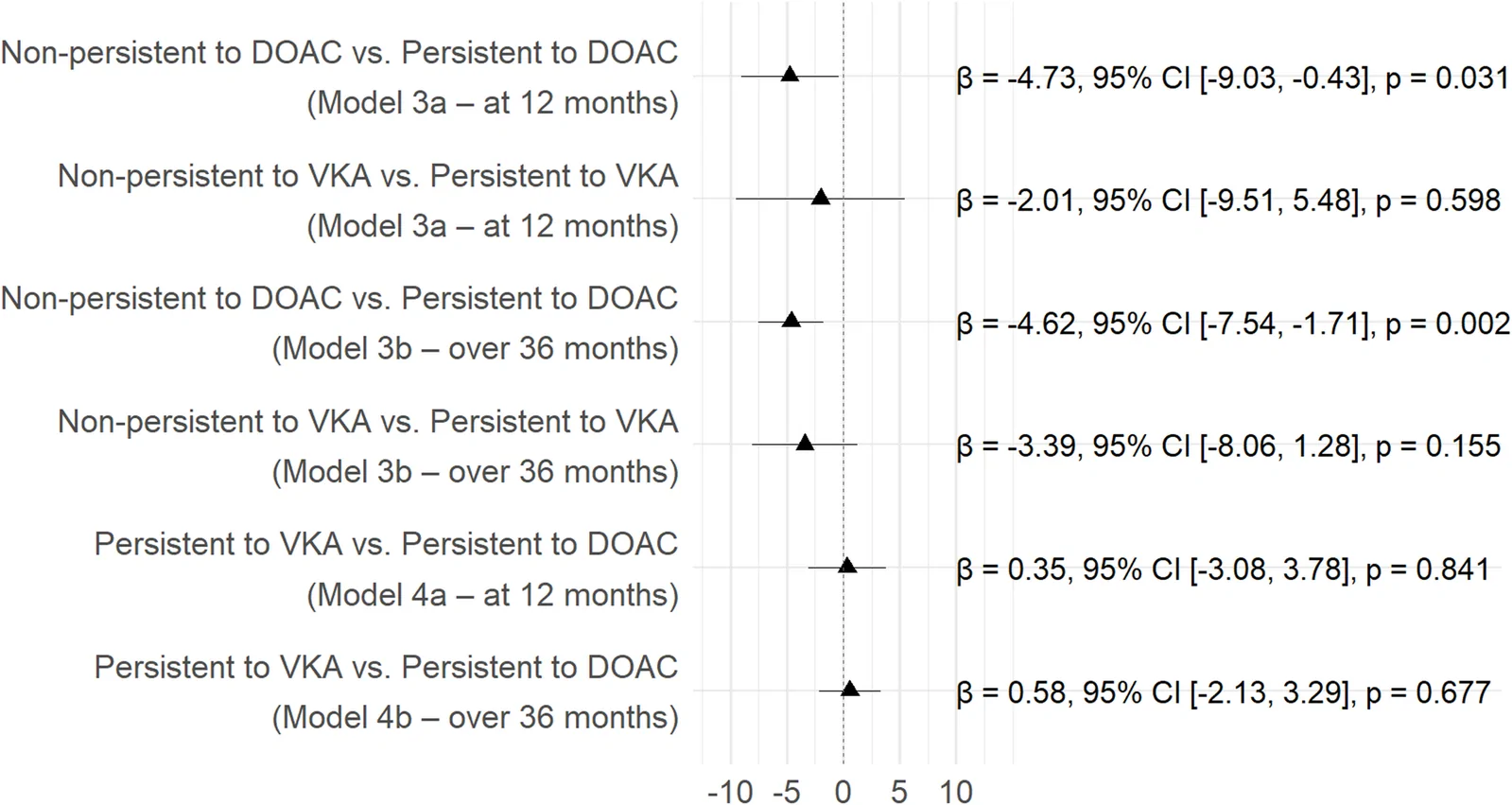
Forest plot with β estimates adjusted for covariates as specified in the respective multivariable models (see Table S9-12) and corresponding 95% confidence intervals on EQ-VAS (▲). *DOAC = direct oral anticoagulants; VKA = Vitamin K antagonists; CI = confidence interval*

Figure 3 displays the adjusted associations between persistence status by type of OAC and EQ-VAS outcomes at 12 and 36 months. Non-persistence to DOAC was statistically significantly associated with lower EQ-VAS scores compared to persistence to DOAC, both at 12 months (β = − 4.73, 95% CI [–9.03, − 0.43], *p* = 0.031; Table S9 – Model 3a) and over 36 months (β = − 4.62, 95% CI [–7.54, − 1.71], *p* = 0.002; Table S11 – Model 3b). Non-persistence to VKA did not result in statistically significant difference in EQ-VAS scores at 12 months (β = -2.01, 95% CI [–9.51, 5.48], *p* = 0.598; Table S9 – Model 3a) and at 36 months (β = − 3.39, 95% CI [–8.06, 1.28], *p* = 0.155; Table S11 – Model 3b). When comparing persistence to VKA versus persistence to DOAC, no statistically significant differences in EQ-VAS scores were observed either at 12 months (see Table S10) or at 36 months (Table S12/Model 4a and 4b).

## Discussion

This post-hoc analysis of the investigator-initiated, multicenter, prospective *Berlin Atrial Fibrillation Registry* examined the association between intake of OAC, persistence to OAC and the type of OAC with HRQoL in stroke survivors with AF over a period of up to 36 months after stroke. Our findings indicate that the absence of OAC intake was associated with lower EQ-VAS at the 12-months follow-up, while non-persistence to OAC was associated with lower EQ-VAS scores at both 12 and 36 months after the index event. This association persisted after adjusting for recurrent strokes or TIA and functional outcomes. We found no evidence of a statistically significant difference in HRQoL between patients who were persistent to DOAC and those who were persistent to VKA.

In a previous analysis of the investigator-initiated, multicenter, prospective, randomized MonDAFIS study, we examined the association between OAC intake and HRQoL in a stroke cohort with a first episode of AF after stroke, reporting higher EQ-Index and EQ-VAS scores at 12 months in patients receiving OAC compared to those patients without OAC [13]. Differences by type of OAC were observed in EQ-VAS scores, suggesting a modest benefit for using a DOAC, whereas no such difference was found for the EQ-Index. Our present study included patients with known AF before stroke as well as patients with a first episode of AF after stroke and identified also an association between OAC intake and EQ-VAS at 12 months. In contrast to the MonDAFIS data, there was no statistically significant association for the EQ-Index, and no differences were observed between DOAC and VKA users in the *Berlin Atrial Fibrillation Registry*. The association between OAC intake and HRQoL was no longer present in the EQ-VAS at 36 months, most likely due to a response shift [17]. A similar mechanism was proposed by Gabilondo et al. to explain the high HRQoL in anticoagulated patients compared to the general population [11].

A recent systematic review on HRQoL including 5,687 patients and comparing DOACs versus VKAs in patients with non-valvular AF found conflicting results [18]. Due to methodological heterogeneity, a meta-analysis was impossible. HRQoL was assessed using disease-specific (*n* = 3) or generic (*n* = 8) instruments. Taken together, four studies favored DOACs, while seven reported no statistically significant difference between treatment groups [18]. Similar findings were reported in a systematic review by Afzal et al., which compared HRQoL in patients with DOACs and warfarin [10]. These findings are in line with the results from the MonDAFIS cohort as well as the present study, both of which found no consistent differences in HRQoL between DOAC and VKA users. Notably, the analysis of the MonDAFIS cohort was limited to OAC intake and type of OAC [13], as treatment persistence – an aspect addressed in the present study – was not assessed. In an observational Spanish AF study (*n* = 501), Varona et al. reported higher HRQoL among patients with well-controlled OAC status compared to those with uncontrolled OAC treatment [19]. Notably, the analyses presented in that study reported HRQoL differences only between persistent and non-persistent DOAC users, while no such differences were found among VKA users. It is also important to note that OAC persistence was defined differently in that study, which may affect the comparability of results.

Several mechanisms may explain that non-persistence to OAC negatively impacts HRQoL over time. First, discontinuation of OAC increases the risk of recurrent ischemic stroke, which may lead to additional neurological impairments and reduced independence [2]. Since recurrent stroke and TIA were accounted for in the models used in our analysis, they are unlikely to explain the observed association between non-persistence and reduced HRQoL. Second, it was reported that OAC therapy per se does not negatively impact HRQoL, but patients’ perceptions of OAC are associated with self-reported HRQoL [20]. Whether this also applies to the *Berlin Atrial Fibrillation Registry* cohort cannot be determined, as reasons for non-persistence were only available for a small subset of registry patients (Table S13). Notably, some registry patients discontinued OAC based on personal preference — cases that, in contrast to medical contraindications, might be preventable. It remains unclear whether the observed association reflects the effect of non-persistence itself or the underlying circumstances that prompted discontinuation. Risk factors associated with an increased bleeding risk — such as hypertension, renal impairment, prior stroke or TIA, and older age — were, with the exception of renal impairment, evenly distributed between patients receiving OAC and those without OAC 12 months after stroke/TIA. Of note, renal impairment was adjusted for in the multivariable models. Third, non-persistence to OAC may account for a higher rate of dementia, affecting HRQoL [3]. Lastly, insufficient family or social support may play a role in undermining both, persistence and psychosocial well-being [21], which was not assessed in the *Berlin Atrial Fibrillation Registry*. Given the observational nature of our study, the association between OAC persistence and HRQoL should not be interpreted causally. Moreover, the direction of this association cannot be determined. While persistent OAC use may contribute to better HRQoL, it is also conceivable that patients with better baseline HRQoL are more likely to remain persistent to long-term-anticoagulation, whereas lower HRQoL, including due to high rates of post-stroke psychiatric comorbidities (kufner) may predispose to lower persistence. Longitudinal studies specifically designed to assess this question would be required to disentangle these effects.

Although statistically significant differences in EQ-VAS were observed, the clinical relevance of these findings remains unclear. One study estimated the minimal clinically important difference in EQ-VAS to be 8.61 using an anchor-based approach and 10.82 using a distribution-based method [22]. Based on these results, the effect sizes observed in our analysis fall below these thresholds. Nevertheless, the consistency of the association across multiple follow-up points for persistence to OAC with EQ-VAS suggests that the impact on patient-perceived health status may be meaningful — especially over longer periods.

Among the strengths of this post hoc study is the use of a prospective, longitudinal design with multiple follow-up points. The EQ-5D questionnaire is one of the most commonly used measures in stroke trials [23] and has the most promising psychometric evidence for use in patients with stroke due to a systematic review [24]. In addition, HRQoL data were available for over 90% of participants at all time points, resulting in a low proportion of missing data. This study adds value by specifically analyzing phenprocoumon, while most prior work examined VKAs in general or focused on warfarin. However, several limitations must be acknowledged. It was not possible to adjust for major bleeding events due to a low number of cases, however, previous evidence suggests no increased ICH risk with DOACs in patients with AF and comparable neurological deficits in DOAC-associated ICH compared with those without OAC (nolte). The extension of the study from 24 to 36 months may have contributed to higher loss to follow-up rates and, consequently, influenced the estimated persistence rates. Although the joint model accounted for mortality during long-term follow-up (12–36 months), it did not address early mortality before the 12-months follow-up. Furthermore, loss to follow-up unrelated to death was not explicitly accounted for, as inverse probability of censoring weighting or other methods to account for informative censoring were not applied. To evaluate potential attrition bias, we compared participants who dropped-out before the first HRQoL assessment at 3-months follow-up (Table S2a). These patients had more severe strokes, which are associated with poorer functional outcomes and HRQoL (function outcome) and a greater burden of comorbidities. Consequently, severely affected patients were likely underrepresented in the longitudinal analyses, which may have introduced survivor and attrition bias. Selection bias by registry design may have led to an underrepresentation of severely affected individuals – individuals who are more likely to be non-persistent and report lower HRQoL. As a result, the associations observed in this study may in fact be underestimated and more pronounced in a broader real-world population. In addition, pre-stroke HRQoL was not available although it is a known predictor of post-stroke HRQoL because the study enrolled patients after the acute stroke. As there is no standardized definition of OAC persistence, our pragmatic definition of persistence (absence of an OAC interruption of more than 7 days) may not fully capture the complexity of treatment patterns, thereby limiting comparability with other studies. Our pragmatic definition may have grouped some patients with a temporary interruption together with those who permanently discontinued treatment. Consequently, some patients classified as non-persistent in our analysis, may nevertheless have had good overall treatment persistence. The relatively small number of patients receiving VKA limited the statistical power of the treatment-specific analyses. Finally, high rates of OAC intake in a German cohort study reflects a high standard of care and may limit the generalizability of the findings to other healthcare settings beyond comparable high-income countries.

## Conclusion

Non-persistence to OAC is associated with lower HRQoL up to 36 months after stroke. Notably, no evidence of a statistically significant difference in HRQoL was observed between patients with persistence to DOAC or VKA use. Future studies should explore the reasons for non-persistence and evaluate interventions to support long-term persistence as non-persistent patients seem to be at risk of a decline in HRQoL.

## Supplementary Information

Below is the link to the electronic supplementary material.

Supplementary Material 1

## Data Availability

Data are available from the authors upon reasonable request.
