# Supplementary material for: Association of oral anticoagulation persistence and health-related quality of life in stroke survivors with atrial fibrillation: long-term results of the Berlin atrial fibrillation registry
Source: Neurol Res Pract. 2026 Sep 16;8(1):78. doi: 10.1186/s42466-026-00534-z (PMC13579717; doi:10.1186/s42466-026-00534-z)
Supplement: Supplementary file 1 [file 42466_2026_534_MOESM1_ESM.docx]

Supplement

Independent variables included in the multivariable linear models

The multivariable linear models (models for EQ-Index and EQ-VAS at 12 months) were adjusted for: EQ-Index/EQ-VAS at 3 months after the index event, age, sex (female/male), years of education (≤ 8, 9-10, ≥ 11 years), National Institutes of Health Stroke Scale (NIHSS) on admission (NIHSS 0, 1-4, ≥ 5), modified Rankin Scale (mRS) at baseline (<3/ ≥ 3), modified Rankin Scale (mRS) at 3 months (<3/ ≥ 3), modified Rankin Scale (mRS) at 12 months (<3/ ≥ 3), type of index event (Ischemic stroke/TIA), intravenous thrombolysis (yes/no), endovascular treatment (yes/no), cardiovascular risk factors, which were either preexisting or diagnosed at baseline: diabetes mellitus (yes/no), arterial hypertension (yes/no), vascular disease defined as coronary heart disease and/or peripheral artery disease and/or prior myocardial infarction (yes/no), renal impairment (yes/no), congestive heart failure (yes/no), prior stroke or TIA to index event (yes/no), and severe adverse events (SAE): SAE within 3 months after stroke (TIA, stroke [including subarachnoid hemorrhage, ischemic and hemorrhagic stroke], major bleeding [including intracranial, non-intracerebral bleeding and extracranial bleeding], myocardial infarction, and peripheral arterial embolism) (yes/no), TIA between 3 and 12 months after index event (yes/no), stroke [including subarachnoid hemorrhage, ischemic and hemorrhagic stroke] between 3 and 12 months after index event (yes/no).

In the multivariable mixed models for time series analysis up to the 36-month follow-up, additional adjustments were made for the following variables, alongside those previously mentioned: TIA between 3 and 36 months after index event (yes/no), stroke [including subarachnoid hemorrhage, ischemic and hemorrhagic stroke] between 3 and 36 months after the index event (yes/no), as well as time (12-, 24-, 36-month follow up) and patient ID as random effect.

**Table S1: Unadjusted HRQoL by SAE***

|  | Available EQ-Index | EQ-Index at follow up | Available EQ-VAS | EQ-VAS at follow-up |
| --- | --- | --- | --- | --- |
|  | N (%) | Mean (SD) | N (%) | Mean (SD) |
| SAE within 3 months after stroke |  |  |  |  |
| yes | 47 (5.5%) | 0.70 (0.32)^1^ | 48 (5.6%) | 56.7 (19.0)^1^ |
| no | 805 (94.5%) | 0.77 (0.27)^1^ | 807 (94.4%) | 63.7 (21.4)^1^ |
| SAE between 3-12 months |  |  |  |  |
| yes | 58 (7.2%) | 0.70 (0.30)^2^ | 58 (7.2%) | 60.9 (18.2)^2^ |
| no | 744 (92.8%) | 0.78 (0.26)^2^ | 744 (92.8%) | 64.2 (20.8)^2^ |
|  |  |  |  |  |
| SAE between 12-24 months |  |  |  |  |
| yes | 35 (5.2%) | 0.69 (0.26)^3^ | 35 (5.1%) | 57.5 (18.5)^3^ |
| no | 643 (94.8%) | 0.78 (0.26)^3^ | 645 (94.4%) | 64.7 (20.6)^3^ |
| SAE between 24-36 months |  |  |  |  |
| yes | 20 (4.8%) | 0.74 (0.23)^4^ | 20 (4.8%) | 53.4 (21.1)^4^ |
| no | 397 (95.2%) | 0.80 (0.25)^4^ | 399 (95.2%) | 66.4 (20.5)^4^ |
|  |  |  |  |  |

^*^ SAE included patients with recurrent TIA, stroke [including subarachnoid hemorrhage, ischemic and hemorrhagic stroke], major bleeding [including intracranial, non-intracerebral bleeding and extracranial bleeding], myocardial infarction, and peripheral arterial embolism

^1^ HRQoL at 3 months follow-up ^2^ HRQoL at 12 months follow-up ^3^ HRQoL at 24 months follow-up ^4^ HRQoL at 36 months follow-up

|  | | **Table S2a – Baseline demographic and clinical characteristics of complete analysis set, study cohort**  **at FU3 and drop-outs until FU3** | | | | | | | | |
| --- | --- | --- | --- | --- | --- | --- | --- | --- | --- | --- |
|  | |  | | Baseline (n=1042) | | FU3 (n=927) | | Drop-outs until FU3 (n=115) | |  |
|  | | **Age at index event in years** | |  | |  | |  | |  |
|  | | Mean (SD) | | 76.2 (9.7) | | 75.9 (9.8) | | 78.8 (8.1) | |  |
|  | | **Sex** | |  | |  | |  | |  |
|  | | Male (n; %) | | 542 (52.0%) | | 482 (52.0%) | | 60 (52.2%) | |  |
|  | | Female (n; %) | | 500 (48.0%) | | 445 (48.0%) | | 55 (47.8%) | |  |
|  | | **Education in years** | |  | |  | |  | |  |
|  | | < 9 (n; %) | | 317 (31.3%) | | 276 (30.5%) | | 41 (38.3%) | |  |
|  | | 9-10 (n; %) | | 426 (42.1%) | | 382 (42.2%) | | 44 (41.1%) | |  |
|  | | > 10 (n; %) | | 269 (26.6%) | | 247 (27.3%) | | 22 (20.6%) | |  |
|  | | **NIHSS score on admission** | |  | |  | |  | |  |
|  | | 0 (n; %) | | 227 (21.8%) | | 214 (23.1%) | | 13 (11.3%) | |  |
|  | | 1-4 (n; %) | | 509 (48.8%) | | 453 (48.9%) | | 56 (48.7%) | |  |
|  | | >4 (n; %) | | 306 (29.4%) | | 260 (28.0%) | | 46 (40.0%) | |  |
|  | | **Modified Rankin scale score at discharge** | |  | |  | |  | |  |
|  | | 0-2 (n; %) | | 540 (52.1%) | | 508 (54.8%) | | 32 (29.4%) | |  |
|  | | > 2 (n; %) | | 496 (47.9%) | | 419 (45.2%) | | 77 (70.6%) | |  |
|  | | **Index stroke** | |  | |  | |  | |  |
|  | | TIA (n; %) | | 251 (24.1%) | | 229 (24.7%) | | 22 (19.1%) | |  |
|  | | Ischemic Stroke (n; %) | | 791 (75.9%) | | 698 (75.3%) | | 93 (80.9%) | |  |
|  | | **Endovascular treatment** (n; %) | | 81 (7.8%) | | 73 (7.9%) | | 8 (7.0%) | |  |
|  | | **Intravenous thrombolysis** (n; %) | | 155 (14.9%) | | 147 (15.9%) | | 8 (7.0%) | |  |
|  | | **Congestive heart failure** (n; %) | | 153 (14.7%) | | 123 (13.3%) | | 30 (26.1%) | |  |
|  | | **Hypertension** (n; %) | | 907 (87.0%) | | 800 (86.3%) | | 107 (93.0%) | |  |
|  | | **Diabetes mellitus** (n; %) | | 294 (28.2%) | | 253 (27.3%) | | 41 (35.7%) | |  |
|  | | **Prior stroke or TIA** (n; %) | | 284 (27.3%) | | 244 (26.3%) | | 40 (34.8%) | |  |
|  | | **Arterial disease** (n; %) | | 296 (28.4%) | | 250 (27.0%) | | 46 (40.0%) | |  |
|  | | **Renal impairment** (n; %) | | 412 (39.6%) | | 344 (37.1%) | | 68 (59.6%) | |  |
|  | | **Type of OAC at discharge** | |  | |  | |  | |  |
|  | | Apixaban (n; %) | | 454 (43.6%) | |  | |  | |  |
|  | Dabigatran (n; %) | | | 136 (13.1%) | |  | |  | |  |
| Edoxaban (n; %) | | | 5 (0.0%) | |  | |  | |  |  |
| Rivaroxaban (n; %) | | | 111 (10.7%) | |  | |  | |  |  |
| Phenprocoumon (n; %) | | | 136 (13.1%) | |  | |  | |  |  |
| No OAC (n; %) | | | 200 (19.2%) | |  | |  | |  |  |

| **Table S2b - Baseline demographic and clinical characteristics for persistent and non-persistent OAC intake** | | | | | | |
| --- | --- | --- | --- | --- | --- | --- |
|  | No OAC at FU12 (N=44) | OAC at FU12 (N=798) | | Persistent at FU12 (N=744) | | Non-persistent at FU12 (N=73) |
| **Age at index event in years** |  |  | |  | |  |
| Mean (SD) | 74.0 (13.8) | 75.5 (9.5) | | 75.5 (9.5) | | 75.7 (11.0) |
| **Sex** |  |  | |  | |  |
| Male (n; %) | 31 (70.5%) | 413 (51.8%) | | 382 (51.3%) | | 46 (63.0%) |
| Female (n; %) | 13 (29.5%) | 385 (48.2%) | | 362 (48.7%) | | 27 (37.0%) |
| **Education in years** |  |  | |  | |  |
| < 9 (n; %) | 13 (30.2%) | 223 (28.6%) | | 210 (28.8%) | | 19 (26.4%) |
| 9-10 (n; %) | 15 (34.9%) | 337 (43.2%) | | 313 (42.9%) | | 33 (45.8%) |
| > 10 (n; %) | 15 (34.9%) | 220 (28.2%) | | 206 (28.3%) | | 20 (27.8%) |
| **NIHSS score on admission** |  |  | |  | |  |
| 0 (n; %) | 15 (34.1%) | 191 (23.9%) | | 176 (23.7%) | | 21 (28.8%) |
| 1-4 (n; %) | 16 (36.4%) | 395 (49.5%) | | 368 (49.5%) | | 33 (45.2%) |
| >4 (n; %) | 13 (29.5%) | 212 (26.6%) | | 200 (26.9%) | | 19 (26.0%) |
| **Modified Rankin scale score at discharge** |  |  | |  | |  |
| 0-2 (n; %) | 26 (59.1%) | 455 (57.0%) | | 427 (57.4%) | | 37 (50.7%) |
| > 2 (n; %) | 18 (40.9%) | 343 (43.0%) | | 317 (42.6%) | | 36 (49.3%) |
| **Index stroke** |  |  | |  | |  |
| TIA (n; %) | 11 (25.0%) | 206 (25.8%) | | 188 (25.3%) | | 20 (27.4%) |
| Ischemic Stroke (n; %) | 33 (75.0%) | 592 (74.2%) | | 556 (74.7%) | | 53 (72.6%) |
| **Endovascular treatment** (n; %) | 3 (6.8%) | 64 (8.0%) | | 63 (8.5%) | | 3 (4.1%) |
| **Intravenous thrombolysis** (n; %) | 5 (11.6%) | 128 (16.1%) | | 121 (16.3%) | | 9 (12.5%) |
| **Congestive heart failure** (n; %) | 9 (20.5%) | 99 (12.4%) | | 92 (12.4%) | | 11 (15.1%) |
| **Hypertension** (n; %) | 38 (86.4%) | 687 (86.1%) | | 639 (85.9%) | | 63 (86.3%) |
| **Diabetes mellitus** (n; %) | 15 (34.1%) | 212 (26.6%) | | 195 (26.2%) | | 24 (32.9%) |
| **Prior stroke or TIA** (n; %) | 9 (20.5%) | 210 (26.3%) | | 191 (25.7%) | | 26 (35.6%) |
| **Arterial disease** (n; %) | 17 (38.6%) | 210 (26.3%) | | 193 (25.9%) | | 25 (34.2%) |
| **Renal impairment** (n; %) | 24 (54.5%) | 279 (35.0%) | | 262 (35.2%) | | 26 (35.6%) |
|  |  |  |  | |  |  |
|  |  |  |  | |  |  |

| **Table S3a: Unadjusted HRQoL up to 36 months after index stroke/TIA** \| \| \| \| \| \| \|; \| \| EQ-Index \| \| \| EQ-VAS \| \|; \| Available data (n) \| Available data on EQ-Index (n) \| Mean \| SD \| Available data on EQ-VAS (n) \| Mean \| SD; At 3 months after index event \| 927 \| 852 \| 0.77 \| 0.3 \| 855 \| 63.3 \| 21.3; - OAC \| \| \| 0.77 \| 0.3 \| \| 63.4 \| 21.2; - No OAC \| \| \| 0.71 \| 0.4 \| \| 61.4 \| 23.2; At 12 months after index event \| 842 \| 802 \| 0.78 \| 0.3 \| 802 \| 64.0 \| 20.6; - OAC \| \| \| 0.78 \| 0.3 \| \| 64.3 \| 20.4; - No OAC \| \| \| 0.76 \| 0.3 \| \| 58.4 \| 24.1; At 24 months after index event \| 749 \| 678 \| 0.78 \| 0.3 \| 680 \| 64.4 \| 20.6; - OAC \| \| \| 0.78 \| 0.3 \| \| 64.4 \| 20.6; - No OAC \| \| \| 0.80 \| 0.3 \| \| 63.5 \| 19.4; At 36 months after index event \| 437 \| 417 \| 0.79 \| 0.3 \| 419 \| 65.8 \| 20.7; - OAC \| \| \| 0.79 \| 0.3 \| \| 65.7 \| 20.7; - No OAC \| \| \| 0.79 \| 0.2 \| \| 66.8 \| 20.0 | | | | | | | |
| --- | --- | --- | --- | --- | --- | --- | --- |
| **Table S3b: Unadjusted HRQoL up to 36 months after index stroke/TIA with transformed EQ-Index** | | | | | | | |
|  |  | EQ-Index (transformed) | | | EQ-VAS | | |
|  | Available data (n) | Available data on EQ-Index (n) | Mean | SD | Available data on EQ-VAS (n) | Mean | SD |
| At 3 months after index event | 927 | 852 | 59.6 | 35.9 | 855 | 63.3 | 21.3 |
| - OAC |  |  | 59.8 | 35.7 |  | 63.4 | 21.2 |
| - No OAC |  |  | 56.8 | 39.3 |  | 61.4 | 23.2 |
| At 12 months after index event | 842 | 802 | 59.1 | 34.2 | 802 | 64.0 | 20.6 |
| - OAC |  |  | 59.1 | 34.2 |  | 64.3 | 20.4 |
| - No OAC |  |  | 59.0 | 33.8 |  | 58.4 | 24.1 |
| At 24 months after index event | 749 | 678 | 59.2 | 34.5 | 680 | 64.4 | 20.6 |
| - OAC |  |  | 58.9 | 34.5 |  | 64.4 | 20.6 |
| - No OAC |  |  | 65.9 | 34.7 |  | 63.5 | 19.4 |
| At 36 months after index event | 437 | 417 | 57.8 | 35.4 | 419 | 65.8 | 20.7 |
| - OAC |  |  | 58.1 | 35.5 |  | 65.7 | 20.7 |
| - No OAC |  |  | 52.1 | 32.4 |  | 66.8 | 20.0 |

| **Table S4: OAC intake yes/no at 12 months (Model 1a)** | | | | | | | |
| --- | --- | --- | --- | --- | --- | --- | --- |
|  | **EQ-Index** | | | **EQ-VAS** | | |  |
| *Predictors* | *Estimates* | *95%-CI* | *p* | *Estimates* | *95%-CI* | *p* |  |
| Intercept | 60.94 | 43.26 – 78.62 | **<0.001** | 45.23 | 32.95 – 57.52 | **<0.001** |  |
| HRQOL at 3 months | 0.44 | 0.38 – 0.50 | **<0.001** | 0.45 | 0.38 – 0.52 | **<0.001** |  |
| Age at index event in years | -0.21 | -0.40 – -0.01 | **0.035** | -0.12 | -0.25 – 0.01 | 0.064 |  |
| Female sex | -4.00 | -7.59 – -0.41 | **0.029** | -1.13 | -3.54 – 1.28 | 0.359 |  |
| Education < 9 years |  |  |  |  |  |  |  |
| Education 9-10 years | -0.69 | -5.05 – 3.67 | 0.755 | -0.21 | -3.16 – 2.74 | 0.889 |  |
| Education > 10 years | 2.25 | -2.53 – 7.03 | 0.356 | 0.18 | -3.05 – 3.41 | 0.914 |  |
| NIHSS score on admission 0 |  |  |  |  |  |  |  |
| NIHSS score on admission 1-4 | -3.94 | -8.61 – 0.73 | 0.098 | -0.22 | -3.37 – 2.93 | 0.890 |  |
| NIHSS score on admission > 4 | -6.01 | -12.33 – 0.30 | 0.062 | 1.39 | -2.86 – 5.65 | 0.521 |  |
| Modified Rankin scale score at baseline > 2 | -1.28 | -5.45 – 2.90 | 0.549 | 0.79 | -1.99 – 3.57 | 0.579 |  |
| Modified Rankin scale score at 3 months > 2 | -0.93 | -5.58 – 3.72 | 0.695 | -0.16 | -3.23 – 2.90 | 0.916 |  |
| Modified Rankin scale score at 12 months > 2 | -21.18 | -25.53 – -16.82 | **<0.001** | -11.98 | -14.85 – -9.11 | **<0.001** |  |
| Index event: TIA |  |  |  |  |  |  |  |
| Index event: Ischemic stroke | 3.32 | -1.32 – 7.96 | 0.160 | 0.45 | -2.68 – 3.58 | 0.779 |  |
| Endovascular treatment | -0.68 | -8.14 – 6.78 | 0.857 | 3.22 | -1.73 – 8.18 | 0.202 |  |
| Intravenous thrombolysis | 2.87 | -2.71 – 8.44 | 0.313 | -2.03 | -5.77 – 1.70 | 0.285 |  |
| Congestive heart failure | -5.30 ^*^ | -10.47 – -0.13 | **0.045** | -0.61 | -4.12 – 2.90 | 0.733 |  |
| Hypertension | 2.37 | -2.70 – 7.44 | 0.359 | -2.43 | -5.86 – 0.99 | 0.164 |  |
| Diabetes mellitus | -2.07 | -6.13 – 1.99 | 0.318 | -0.79 | -3.52 – 1.94 | 0.570 |  |
| Prior stroke or TIA | 1.63 | -2.46 – 5.72 | 0.435 | 1.08 | -1.66 – 3.82 | 0.440 |  |
| Vascular disease | -1.18 | -5.24 – 2.87 | 0.567 | -0.27 | -3.00 – 2.45 | 0.844 |  |
| Renal impairment | -1.89 | -5.71 – 1.92 | 0.330 | 0.75 | -1.82 – 3.33 | 0.565 |  |
| SAE within 3 months after stroke | 6.83 | -0.64 – 14.31 | 0.073 | 0.94 | -4.05 – 5.92 | 0.712 |  |
| SAE: TIA between 3-12 months | 2.84 | -10.29 – 15.97 | 0.671 | -0.49 | -9.31 – 8.34 | 0.914 |  |
| SAE: Stroke between 3-12 months | -11.31 | -20.39 – -2.23 | **0.015** | 0.51 | -5.62 – 6.64 | 0.870 |  |
| OAC intake | -0.25 | -8.42 – 7.91 | 0.952 | 6.70 | 1.20 – 12.20 | **0.017** |  |
| Observations | 729 | | | 731 | | |  |
| R^2^ / R^2^ adjusted | 0.540 / 0.524 | | | 0.427 / 0.408 | | |  |

| **Table S5: Persistence to OAC at 12 months (Model 2a)** | | | | | | |
| --- | --- | --- | --- | --- | --- | --- |
|  | **EQ-Index** | | | **EQ-VAS** | | |
| *Predictors* | *Estimates* | *95%-CI* | *p* | *Estimates* | *95%-CI* | *p* |
| Intercept | 60.64 | 43.75 – 77.54 | **<0.001** | 49.85 | 38.15 – 61.55 | **<0.001** |
| HRQOL at 3 months | 0.44 | 0.37 – 0.50 | **<0.001** | 0.44 | 0.38 – 0.51 | **<0.001** |
| Age at index event in years | -0.21 | -0.41 – -0.01 | **0.035** | -0.09 | -0.22 – 0.04 | 0.173 |
| Female sex | -3.88 | -7.53 – -0.23 | **0.037** | -1.34 | -3.78 – 1.09 | 0.279 |
| Education < 9 years |  |  |  |  |  |  |
| Education 9-10 years | -0.33 | -4.76 – 4.09 | 0.882 | 0.30 | -2.68 – 3.28 | 0.841 |
| Education > 10 years | 2.48 | -2.39 – 7.34 | 0.318 | 0.42 | -2.85 – 3.69 | 0.801 |
| NIHSS score on admission 0 |  |  |  |  |  |  |
| NIHSS score on admission 1-4 | -4.25 | -9.02 – 0.51 | 0.080 | -0.57 | -3.77 – 2.62 | 0.724 |
| NIHSS score on admission > 4 | -6.05 | -12.50 – 0.39 | 0.066 | 0.98 | -3.34 – 5.29 | 0.658 |
| Modified Rankin scale score at baseline > 2 | -1.36 | -5.62 – 2.90 | 0.530 | 0.81 | -2.00 – 3.63 | 0.570 |
| Modified Rankin scale score at 3 months > 2 | -1.22 | -5.97 – 3.53 | 0.615 | -0.13 | -3.23 – 2.96 | 0.932 |
| Modified Rankin scale score at 12 months > 2 | -21.42 | -25.82 – -17.02 | **<0.001** | -12.06 | -14.94 – -9.18 | **<0.001** |
| Index event: TIA |  |  |  |  |  |  |
| Index event: Ischemic stroke | 3.68 | -1.08 – 8.43 | 0.130 | 0.75 | -2.44 – 3.95 | 0.643 |
| Endovascular treatment | 0.28 | -7.26 – 7.81 | 0.942 | 3.49 | -1.49 – 8.47 | 0.169 |
| Intravenous thrombolysis | 3.03 | -2.57 – 8.64 | 0.289 | -1.92 | -5.66 – 1.82 | 0.314 |
| Congestive heart failure | -4.54 | -9.80 – 0.73 | 0.091 | -0.69 | -4.24 – 2.87 | 0.705 |
| Hypertension | 2.68 | -2.48 – 7.84 | 0.308 | -2.39 | -5.85 – 1.08 | 0.177 |
| Diabetes mellitus | -2.07 | -6.20 – 2.06 | 0.326 | -0.42 | -3.18 – 2.35 | 0.767 |
| Prior stroke or TIA | 1.66 | -2.46 – 5.78 | 0.429 | 1.32 | -1.42 – 4.06 | 0.346 |
| Vascular disease | -1.24 | -5.35 – 2.87 | 0.554 | -0.04 | -2.79 – 2.71 | 0.976 |
| Renal impairment | -1.52 | -5.39 – 2.36 | 0.442 | 0.75 | -1.84 – 3.35 | 0.569 |
| SAE within 3 months after stroke | 6.48 | -1.12 – 14.09 | 0.094 | 0.57 | -4.47 – 5.61 | 0.825 |
| SAE: TIA between 3-12 months | 2.78 | -10.38 – 15.94 | 0.678 | -0.36 | -9.17 – 8.44 | 0.935 |
| SAE: Stroke between 3-12 months | -11.41 | -20.53 – -2.30 | **0.014** | 0.61 | -5.51 – 6.73 | 0.844 |
| Non-persistent to OAC | 1.14 | -5.28 – 7.55 | 0.728 | -6.25 | -10.55 – -1.95 | **0.004** |
| Observations | 714 | | | 716 | | |
| R^2^ / R^2^ adjusted | 0.539 / 0.523 | | | 0.427 / 0.408 | | |

| **Table S6: OAC intake yes/no over 36 months (Model 1b)** | | | | | | | | | |  |
| --- | --- | --- | --- | --- | --- | --- | --- | --- | --- | --- |
|  | **EQ-Index** | | | **EQ-VAS** | | | | | |  |
| *Predictors* | *Estimates* | *95%-CI* | *p* | *Estimates* | *95%-CI* | | *p* | |  |  |
| Intercept | 64.49 | 49.45 – 79.53 | **<0.001** | 55.58 | 46.00 – 65.16 | | **<0.001** | |  |  |
| Follow-up timepoint | -0.00 | -0.01 – 0.00 | 0.084 | -0.00 | -0.00 – 0.00 | | 0.569 | |  |  |
| HRQOL at 3 months | 0.44 | 0.39 – 0.50 | **<0.001** | 0.42 | 0.37 – 0.48 | | **<0.001** | |  |  |
| Age at index event in years | -0.27 | -0.43 – -0.10 | **0.002** | -0.15 | -0.25 – -0.04 | | **0.007** | |  |  |
| Female sex | -4.31 | -7.41 – -1.21 | **0.006** | -1.52 | -3.49 – 0.45 | | 0.129 | |  |  |
| Education < 9 years |  |  |  |  |  | |  | |  |  |
| Education 9-10 years | 0.58 | -3.21 – 4.37 | 0.765 | -0.33 | -2.76 – 2.11 | | 0.792 | |  |  |
| Education > 10 years | 2.67 | -1.45 – 6.80 | 0.204 | 1.24 | -1.40 – 3.88 | | 0.358 | |  |  |
| NIHSS score on admission 0 |  |  |  |  |  | |  | |  |  |
| NIHSS score on admission 1-4 | -3.42 | -7.43 – 0.59 | 0.095 | 0.08 | -2.48 – 2.63 | | 0.953 | |  |  |
| NIHSS score on admission > 4 | -4.49 | -9.93 – 0.96 | 0.106 | 2.55 | -0.93 – 6.03 | | 0.151 | |  |  |
| Modified Rankin scale score at baseline > 2 | -0.96 | -4.57 – 2.66 | 0.604 | -0.77 | -3.06 – 1.51 | | 0.508 | |  |  |
| Modified Rankin scale score at 3 months > 2 | -2.17 | -6.11 – 1.76 | 0.279 | -1.18 | -3.61 – 1.26 | | 0.342 | |  |  |
| Modified Rankin scale score at 12, 24 or 36 months > 2 | -17.64 | -20.41 – -14.87 | **<0.001** | -10.59 | -12.38 – -8.79 | | **<0.001** | |  |  |
| Index event: TIA |  |  |  |  |  | |  | |  |  |
| Index event: Ischemic stroke | 2.31 | -1.68 – 6.30 | 0.257 | 0.74 | -1.81 – 3.28 | | 0.570 | |  |  |
| Endovascular treatment | 3.13 | -3.26 – 9.53 | 0.336 | 2.28 | -1.74 – 6.29 | | 0.267 | |  |  |
| Intravenous thrombolysis | 1.63 | -3.13 – 6.40 | 0.501 | -2.92 | -5.94 – 0.09 | | 0.057 | |  |  |
| Congestive heart failure | -1.59 | -6.11 – 2.93 | 0.490 | 1.00 | -1.93 – 3.92 | | 0.504 | |  |  |
| Hypertension | 1.57 | -2.79 – 5.94 | 0.480 | -2.78 | -5.56 – -0.01 | | **0.050** | |  |  |
| Diabetes mellitus | -1.34 | -4.87 – 2.19 | 0.457 | -0.73 | -2.98 – 1.52 | | 0.525 | |  |  |
| Prior stroke or TIA | 0.04 | -3.49 – 3.56 | 0.984 | 0.90 | -1.33 – 3.13 | | 0.428 | |  |  |
| Vascular disease | -2.31 | -5.80 – 1.18 | 0.195 | 0.35 | -1.88 – 2.58 | | 0.758 | |  |  |
| Renal impairment | -1.83 | -5.13 – 1.47 | 0.277 | 0.16 | -1.95 – 2.27 | | 0.881 | |  |  |
| SAE within 3 months after stroke | 1.87 | -4.57 – 8.31 | 0.569 | 3.28 | -0.80 – 7.37 | | 0.115 | |  |  |
| SAE: TIA between 3-36 months | 4.67 | -2.96 – 12.29 | 0.230 | 0.65 | -4.10 – 5.40 | | 0.789 | |  |  |
| SAE: Stroke between 3-36 months | -6.35 | -12.42 – -0.29 | **0.040** | -3.26 | -7.11 – 0.59 | | 0.097 | |  |  |
| No OAC intake |  |  |  |  |  | |  | |  |  |
| OAC intake | 0.23 | -6.06 – 6.51 | 0.944 | -3.67 | -7.72 – 0.37 | | 0.075 | |  |  |
| ICC | 0.46 | | | 0.45 | | | | | |  |
| N | 745 | | | 746 | | | | | |  |
| Observations | 1730 | | | 1739 | | | | | |  |
| Marginal R^2^ / Conditional R^2^ | 0.476 / 0.715 | | | 0.400 / 0.669 | | | | | |  |
|  | |  | | |  |  | |  | | |

| **Table S7: Persistence to OAC over 36 months (Model 2b)** | | | | | | | | | |  |
| --- | --- | --- | --- | --- | --- | --- | --- | --- | --- | --- |
|  | **EQ-Index** | | | **EQ-VAS** | | | | | |  |
| *Predictors* | *Estimates* | *95%-CI* | *p* | *Estimates* | *95%-CI* | | *p* | |  |  |
| Intercept | 64.63 | 49.88 – 79.39 | **<0.001** | 54.21 | 44.63 – 63.79 | | **<0.001** | |  |  |
| Follow-up timepoint | -0.00 | -0.01 – 0.00 | 0.095 | -0.00 | -0.00 – 0.00 | | 0.821 | |  |  |
| HRQOL at 3 months | 0.44 | 0.39 – 0.50 | **<0.001** | 0.41 | 0.36 – 0.47 | | **<0.001** | |  |  |
| Age at index event in years | -0.27 | -0.44 – -0.10 | **0.002** | -0.13 | -0.23 – -0.02 | | **0.021** | |  |  |
| Female sex | -4.29 | -7.44 – -1.14 | **0.008** | -1.68 | -3.65 – 0.30 | | 0.096 | |  |  |
| Education < 9 years |  |  |  |  |  | |  | |  |  |
| Education 9-10 years | 0.96 | -2.89 – 4.81 | 0.624 | 0.19 | -2.25 – 2.63 | | 0.878 | |  |  |
| Education > 10 years | 3.04 | -1.17 – 7.25 | 0.156 | 1.76 | -0.89 – 4.41 | | 0.193 | |  |  |
| NIHSS score on admission 0 |  |  |  |  |  | |  | |  |  |
| NIHSS score on admission 1-4 | -3.86 | -7.96 – 0.24 | 0.065 | -0.28 | -2.86 – 2.29 | | 0.828 | |  |  |
| NIHSS score on admission > 4 | -4.71 | -10.28 – 0.86 | 0.098 | 2.18 | -1.33 – 5.69 | | 0.223 | |  |  |
| Modified Rankin scale score at baseline > 2 | -0.95 | -4.64 – 2.74 | 0.613 | -0.65 | -2.95 – 1.65 | | 0.578 | |  |  |
| Modified Rankin scale score at 3 months > 2 | -2.37 | -6.39 – 1.65 | 0.247 | -1.14 | -3.58 – 1.30 | | 0.360 | |  |  |
| Modified Rankin scale score at 12, 24 or 36 months > 2 | -17.52 | -20.31 – -14.74 | **<0.001** | -10.84 | -12.64 – -9.03 | | **<0.001** | |  |  |
| Index event: TIA |  |  |  |  |  | |  | |  |  |
| Index event: Ischemic stroke | 2.52 | -1.57 – 6.62 | 0.227 | 0.84 | -1.73 – 3.41 | | 0.520 | |  |  |
| Endovascular treatment | 3.50 | -2.98 – 9.98 | 0.290 | 2.63 | -1.39 – 6.64 | | 0.199 | |  |  |
| Intravenous thrombolysis | 1.67 | -3.14 – 6.48 | 0.495 | -2.92 | -5.92 – 0.08 | | 0.057 | |  |  |
| Congestive heart failure | -1.06 | -5.67 – 3.55 | 0.651 | 1.14 | -1.81 – 4.08 | | 0.448 | |  |  |
| Hypertension | 1.80 | -2.65 – 6.25 | 0.427 | -2.72 | -5.50 – 0.07 | | 0.056 | |  |  |
| Diabetes mellitus | -1.16 | -4.75 – 2.44 | 0.529 | -0.29 | -2.56 – 1.97 | | 0.798 | |  |  |
| Prior stroke or TIA | 0.16 | -3.39 – 3.72 | 0.928 | 1.02 | -1.20 – 3.24 | | 0.368 | |  |  |
| Vascular disease | -2.17 | -5.72 – 1.38 | 0.231 | 0.62 | -1.62 – 2.85 | | 0.589 | |  |  |
| Renal impairment | -1.73 | -5.09 – 1.62 | 0.310 | 0.22 | -1.89 – 2.33 | | 0.840 | |  |  |
| SAE within 3 months after stroke | 1.57 | -4.97 – 8.12 | 0.637 | 3.09 | -1.00 – 7.18 | | 0.139 | |  |  |
| SAE: TIA between 3-36 months | 4.62 | -3.03 – 12.27 | 0.236 | 0.54 | -4.18 – 5.25 | | 0.823 | |  |  |
| SAE: Stroke between 3-36 months | -6.02 | -12.13 – 0.09 | 0.054 | -2.66 | -6.51 – 1.18 | | 0.174 | |  |  |
| Persistent to OAC |  |  |  |  |  | |  | |  |  |
| Non-persistent to OAC | -2.01 | -6.52 – 2.50 | 0.382 | -5.39 | -8.25 – -2.54 | | **<0.001** | |  |  |
| ICC | 0.47 | | | 0.44 | | | | | |  |
| N | 729 | | | 730 | | | | | |  |
| Observations | 1700 | | | 1709 | | | | | |  |
| Marginal R^2^ / Conditional R^2^ | 0.475 / 0.724 | | | 0.404 / 0.664 | | | | | |  |
|  | |  | | |  |  | |  | | |

**Table S8: Estimates of joint models focusing on OAC variables**

|  | EQ-Index | | EQ-VAS | |
| --- | --- | --- | --- | --- |
|  | Estimates | 95%-CI | Estimates | 95%-CI |
| **Model 5** |  |  |  |  |
| No OAC intake |  |  |  |  |
| OAC intake | 0.40 | -5.48 – 6.36 | -3.61 | -7.46 – 0.20 |
| **Model 6** |  |  |  |  |
| Persistent to OAC |  |  |  |  |
| Non-persistent to OAC | -1.99 | -6.65 – 2.25 | - 5.35 | -7.99 – -2.63 |
| **Model 7** |  |  |  |  |
| Persistent to VKA |  |  |  |  |
| Non-persistent to VKA |  |  | -3.84 | -7.93 – 0.37 |
| No intake of OAC |  |  | -2.38 | -6.40 – 1.68 |
| Persistent to DOAC |  |  |  |  |
| Non-persistent to DOAC |  |  | -4.52 | -7.24 – -1.82 |
| No intake of OAC |  |  | -2.60 | -7.12 – 1.89 |
| **Model 8** |  |  |  |  |
| Persistent to DOAC |  |  |  |  |
| Persistent to VKA |  |  | 0.72 | -1.88 – 3.29 |
| No intake of OAC |  |  | -3.77 | -7.15 – -0.30 |

| **Table S9: Persistence to VKA and DOAC at 12 months (Model 3a)** | | | |
| --- | --- | --- | --- |
|  | **EQ-VAS** | | |
| *Predictors* | *Estimates* | *95%-CI* | *p* |
| Intercept | 50.98 | 37.40 – 64.57 | **<0.001** |
| HRQOL at 3 months | 0.45 | 0.38 – 0.51 | **<0.001** |
| Age at index event in years | -0.10 | -0.23 – 0.04 | 0.157 |
| Female sex | -1.21 | -3.66 – 1.24 | 0.332 |
| Education < 9 years |  |  |  |
| Education 9-10 years | 0.28 | -2.71 – 3.28 | 0.852 |
| Education > 10 years | 0.48 | -2.82 – 3.79 | 0.774 |
| NIHSS score on admission 0 |  |  |  |
| NIHSS score on admission 1-4 | -0.51 | -3.71 – 2.70 | 0.757 |
| NIHSS score on admission > 4 | 1.01 | -3.33 – 5.34 | 0.649 |
| Modified Rankin scale score at baseline > 2 | 0.60 | -2.23 – 3.44 | 0.675 |
| Modified Rankin scale score at 3 months > 2 | -0.15 | -3.27 – 2.96 | 0.923 |
| Modified Rankin scale score at 12 months > 2 | -11.93 | -14.83 – -9.03 | **<0.001** |
| Index event: TIA |  |  |  |
| Index event: Ischemic stroke | 0.86 | -2.35 – 4.06 | 0.601 |
| Endovascular treatment | 3.42 | -1.60 – 8.44 | 0.182 |
| Intravenous thrombolysis | -1.56 | -5.35 – 2.23 | 0.419 |
| Congestive heart failure | -0.68 | -4.25 – 2.90 | 0.710 |
| Hypertension | -2.49 | -5.98 – 1.00 | 0.162 |
| Diabetes mellitus | -0.34 | -3.12 – 2.44 | 0.811 |
| Prior stroke or TIA | 1.21 | -1.55 – 3.97 | 0.389 |
| Vascular disease | -0.06 | -2.82 – 2.70 | 0.964 |
| Renal impairment | 0.79 | -1.82 – 3.40 | 0.554 |
| SAE within 3 months after stroke | 0.67 | -4.40 – 5.73 | 0.796 |
| SAE: TIA between 3-12 months | -0.01 | -8.87 – 8.85 | 0.998 |
| SAE: Stroke between 3-12 months | 0.28 | -5.86 – 6.41 | 0.930 |
| Persistent to DOAC |  |  |  |
| Non-persistent to DOAC | -4.73 | -9.03 – -0.43 | **0.031** |
| No intake of DOAC | -0.40 | -7.73 – 6.92 | 0.915 |
| Persistent to VKA |  |  |  |
| Non-persistent to VKA | -2.01 | -9.51 – 5.48 | 0.598 |
| No intake of VKA | -1.12 | -7.86 – 5.62 | 0.744 |
| Observations | 716 | |  |
| R^2^ / R^2^ adjusted | 0.425 / 0.403 | |  |
|  | | |  |

| **Table S10: Head-to-head comparison VKA/DOAC persistence at 12 months (Model 4a)** | | | |
| --- | --- | --- | --- |
|  | **EQ-VAS** | | |
| *Predictors* | *Estimates* | *95%-CI* | *p* |
| Intercept | 49.46 | 37.50 – 61.41 | **<0.001** |
| HRQOL at 3 months | 0.44 | 0.38 – 0.51 | **<0.001** |
| Age at index event in years | -0.09 | -0.23 – 0.04 | 0.166 |
| Female sex | -1.28 | -3.72 – 1.17 | 0.305 |
| Education < 9 years |  |  |  |
| Education 9-10 years | 0.18 | -2.81 – 3.18 | 0.904 |
| Education > 10 years | 0.33 | -2.95 – 3.62 | 0.842 |
| NIHSS score on admission 0 |  |  |  |
| NIHSS score on admission 1-4 | -0.43 | -3.64 – 2.77 | 0.791 |
| NIHSS score on admission > 4 | 1.09 | -3.24 – 5.43 | 0.621 |
| Modified Rankin scale score at baseline > 2 | 0.63 | -2.20 – 3.46 | 0.664 |
| Modified Rankin scale score at 3 months > 2 | -0.08 | -3.19 – 3.04 | 0.962 |
| Modified Rankin scale score at 12 months > 2 | -12.08 | -14.97 – -9.19 | **<0.001** |
| Index event: TIA |  |  |  |
| Index event: Ischemic stroke | 0.80 | -2.41 – 4.00 | 0.626 |
| Endovascular treatment | 3.44 | -1.58 – 8.46 | 0.178 |
| Intravenous thrombolysis | -1.86 | -5.62 – 1.89 | 0.330 |
| Congestive heart failure | -0.65 | -4.23 – 2.92 | 0.720 |
| Hypertension | -2.33 | -5.81 – 1.15 | 0.189 |
| Diabetes mellitus | -0.47 | -3.25 – 2.30 | 0.737 |
| Prior stroke or TIA | 1.28 | -1.47 – 4.04 | 0.361 |
| Vascular disease | -0.12 | -2.87 – 2.64 | 0.935 |
| Renal impairment | 0.73 | -1.88 – 3.34 | 0.584 |
| SAE within 3 months after stroke | 0.56 | -4.50 – 5.61 | 0.829 |
| SAE: TIA between 3-12 months | -0.26 | -9.10 – 8.58 | 0.953 |
| SAE: Stroke between 3-12 months | 0.11 | -6.01 – 6.24 | 0.971 |
| Persistent to VKA |  |  |  |
| Persistent to DOAC | 0.35 | -3.08 – 3.78 | 0.841 |
| Non-persistent | -4.08 | -9.17 – 1.01 | 0.116 |
| Observations | 716 | | |
| R^2^ / R^2^ adjusted | 0.424 / 0.404 | | |

| **Table S11: Persistence to VKA and DOAC over 36 months (Model 3b)** | | | |
| --- | --- | --- | --- |
|  | **EQ-VAS** | | |
| *Predictors* | *Estimates* | *95%-CI* | *p* |
| Intercept | 56.60 | 45.99 – 67.21 | **<0.001** |
| Follow-up timepoint | -0.00 | -0.00 – 0.00 | 0.857 |
| HRQOL at 3 months | 0.41 | 0.36 – 0.47 | **<0.001** |
| Age at index event in years | -0.13 | -0.24 – -0.02 | **0.019** |
| Female sex | -1.59 | -3.57 – 0.39 | 0.116 |
| Education < 9 years |  |  |  |
| Education 9-10 years | 0.07 | -2.39 – 2.53 | 0.955 |
| Education > 10 years | 1.66 | -1.02 – 4.33 | 0.225 |
| NIHSS score on admission 0 |  |  |  |
| NIHSS score on admission 1-4 | -0.20 | -2.78 – 2.38 | 0.878 |
| NIHSS score on admission > 4 | 2.30 | -1.22 – 5.83 | 0.200 |
| Modified Rankin scale score at baseline > 2 | -0.86 | -3.18 – 1.45 | 0.463 |
| Modified Rankin scale score at 3 months > 2 | -1.15 | -3.60 – 1.30 | 0.357 |
| Modified Rankin scale score at 12, 24 or 36 months >2 | -10.77 | -12.59 – -8.96 | **<0.001** |
| Index event: TIA |  |  |  |
| Index event: Ischemic stroke | 0.83 | -1.76 – 3.42 | 0.529 |
| Endovascular treatment | 2.44 | -1.61 – 6.48 | 0.237 |
| Intravenous thrombolysis | -2.70 | -5.72 – 0.33 | 0.081 |
| Congestive heart failure | 1.16 | -1.81 – 4.12 | 0.444 |
| Hypertension | -2.79 | -5.59 – 0.01 | 0.051 |
| Diabetes mellitus | -0.23 | -2.51 – 2.05 | 0.842 |
| Prior stroke or TIA | 0.92 | -1.31 – 3.15 | 0.418 |
| Vascular disease | 0.61 | -1.63 – 2.86 | 0.591 |
| Renal impairment | 0.27 | -1.85 – 2.40 | 0.802 |
| SAE within 3 months after stroke | 3.13 | -0.98 – 7.23 | 0.135 |
| SAE: TIA between 3-36 months | 0.80 | -3.93 – 5.53 | 0.741 |
| SAE: Stroke between 3-36 months | -2.78 | -6.63 – 1.08 | 0.157 |
| Persistent to VKA |  |  |  |
| Non-persistent to VKA | -3.39 | -8.06 – 1.28 | 0.155 |
| No intake of OAC | -2.01 | -6.63 – 2.61 | 0.393 |
| Persistent to DOAC |  |  |  |
| Non-persistent to DOAC | -4.62 | -7.54 – -1.71 | **0.002** |
| No intake of OAC | -2.00 | -6.98 – 2.99 | 0.432 |
| ICC | 0.44 | | |
| N | 730 | | |
| Observations | 1709 | | |
| Marginal R^2^ / Conditional R^2^ | 0.402 / 0.666 | | |
|  | | |  |

| **Table S12: Head-to-head comparison VKA/DOAC persistence over 36 months (Model 4b)** | | | |
| --- | --- | --- | --- |
|  | **EQ-VAS** | | |
| *Predictors* | *Estimates* | *95%-CI* | *p* |
| Intercept | 53.81 | 44.05 – 63.57 | **<0.001** |
| Follow-up timepoint | -0.00 | -0.00 – 0.00 | 0.794 |
| HRQOL at 3 months | 0.42 | 0.36 – 0.47 | **<0.001** |
| Age at index event in years | -0.13 | -0.23 – -0.02 | **0.020** |
| Female sex | -1.62 | -3.60 – 0.36 | 0.108 |
| Education < 9 years |  |  |  |
| Education 9-10 years | 0.04 | -2.40 – 2.49 | 0.973 |
| Education > 10 years | 1.63 | -1.02 – 4.29 | 0.228 |
| NIHSS score on admission 0 |  |  |  |
| NIHSS score on admission 1-4 | -0.20 | -2.77 – 2.38 | 0.880 |
| NIHSS score on admission > 4 | 2.28 | -1.24 – 5.80 | 0.204 |
| Modified Rankin scale score at baseline > 2 | -0.80 | -3.11 – 1.50 | 0.495 |
| Modified Rankin scale score at 3 months > 2 | -1.11 | -3.56 – 1.33 | 0.371 |
| Modified Rankin scale score at 12, 24 or 36 months > 2 | -10.84 | -12.65 – -9.03 | **<0.001** |
| Index event: TIA |  |  |  |
| Index event: Ischemic stroke | 0.84 | -1.74 – 3.42 | 0.521 |
| Endovascular treatment | 2.50 | -1.54 – 6.53 | 0.224 |
| Intravenous thrombolysis | -2.84 | -5.84 – 0.17 | 0.065 |
| Congestive heart failure | 1.20 | -1.76 – 4.15 | 0.428 |
| Hypertension | -2.74 | -5.53 – 0.05 | 0.055 |
| Diabetes mellitus | -0.32 | -2.59 – 1.95 | 0.779 |
| Prior stroke or TIA | 0.99 | -1.24 – 3.22 | 0.383 |
| Vascular disease | 0.58 | -1.66 – 2.82 | 0.609 |
| Renal impairment | 0.18 | -1.94 – 2.29 | 0.870 |
| SAE within 3 months after stroke | 3.06 | -1.03 – 7.16 | 0.143 |
| SAE: TIA between 3-36 months | 0.72 | -4.00 – 5.45 | 0.764 |
| SAE: Stroke between 3-36 months | -2.90 | -6.74 – 0.94 | 0.139 |
| Persistent to DOAC |  |  |  |
| Persistent to VKA | 0.58 | -2.13 – 3.29 | 0.677 |
| No intake of OAC | -3.88 | -7.44 – -0.33 | **0.032** |
| ICC | 0.44 | | |
| N | 730 | | |
| Observations | 1709 | | |
| Marginal R^2^ / Conditional R^2^ | 0.402 / 0.665 | | |
|  | | |  |

| **Table S13: Reasons for treatment discontinuation of OAC in non-persistent patients** | | | |
| --- | --- | --- | --- |
|  | **Follow-Up-Visit** | | |
| Reason^1^ (n) | 12-months* | 24-months** | 36-months*** |
| Unknown | 2 | 0 | 0 |
| Recurrent Stroke | 2 | 1 | 2 |
| Bleeding | 5 | 2 | 2 |
| Myocardial infarction | 0 | 0 | 0 |
| Renal impairment | 3 | 1 | 1 |
| Dysphagia | 0 | 0 | 0 |
| Irregular intake | 0 | 0 | 0 |
| Patient preference | 2 | 6 | 0 |
| Side effects | 3 | 0 | 1 |
| Physician-recommended OAC-discontinuation | 12 | 8 | 2 |
| Other | 9 | 6 | 2 |
| ^1^ Multiple responses allowed * Available information of discontinuation in patients with non-persistence, n (%): 30/73 (41.1%)  **Available information of discontinuation in patients with non-persistence, n (%): 21/83 (25.3%)  ***Available information of discontinuation in patients with non-persistence, n (%): 10/54 (18.5%) | | | |
